# Ningetinib, a novel FLT3 inhibitor, overcomes secondary drug resistance in acute myeloid leukemia

**DOI:** 10.1186/s12964-024-01729-0

**Published:** 2024-07-08

**Authors:** Chuhong Hu, Yvyin Zhang, Jie Yang, Yanli Xu, Tingfen Deng, Yumiao Li, Shilin Xu, Shunqing Wang, Peihong Wang

**Affiliations:** 1 Department of Hematology, the Second Affiliated Hospital, School of Medicine, South China University of Technology, Guangzhou, 510180 Guangdong China; 2 Department of Hematology, Guangzhou First People’s Hospital, Guangzhou, 510180 Guangdong China; 3 Department of Hematology, Guangdong Provincial People’s Hospital (Guangdong Academy of Medical Sciences), Southern Medical University, Guangzhou, 510080 Guangdong China

**Keywords:** Ningetinib, Acute myeloid leukemia, FLT3-ITD, Gilteritinib, Quizartinib, FLT3 inhibitor resistance

## Abstract

**Background:**

FMS-like tyrosine kinase 3 internal tandem duplication (FLT3-ITD) is a common mutation type in acute myeloid leukemia (AML) and is usually associated with poor patient prognosis. With advancements in molecular diagnostics and the development of tyrosine kinase inhibitors (TKI), the overall survival (OS) of AML patients with FLT3-ITD mutations has been prolonged to some extent, but relapse and drug resistance are still substantial challenges. Ningetinib is a novel TKI against various kinases in relation to tumour pathogenesis and is undergoing clinical trials of lung cancer. In this study, we explored the antitumor activity of ningetinib against AML with FLT3 mutations both in vivo and in vitro.

**Methods:**

Cell proliferation assays were performed in AML cell lines and Ba/F3 cells expressing various FLT3 mutations to validate the antileukemic activity of ningetinib in vitro. Immunoblot assays were used to verify the effect of ningetinib on the FLT3 protein and downstream pathways. Molecular docking and CETSA were used to validate the interaction of ningetinib with target proteins. The survival benefit of ningetinib in vivo was assessed in Ba/F3-FLT3-ITD-, MOLM13, Ba/F3-FLT3-ITD-F691L-, MOLM13-FLT3-ITD-F691L-induced leukemia mouse models. We also used patient-derived primary cells to determine the efficacy of ningetinib.

**Results:**

Ningetinib inhibited cell proliferation, blocked the cell cycle, induced apoptosis and bound FLT3 to inhibit its downstream signaling pathways, including the STAT5, AKT and ERK pathways, in FLT3-ITD AML cell lines. In the mouse models with FLT3-ITD and FLT3-ITD-F691L mutation, ningetinib showed superior anti-leukemia activity to existing clinical drugs gilteritinib and quizartinib, significantly prolongating the survival of mice. In addition, ningetinib exhibited activity against patient-derived primary cells harboring FLT3-ITD mutations.

**Conclusion:**

Overall, our study confirmed the therapeutic role of ningetinib in AML with FLT3-ITD mutations, providing a potential new option for clinically resistant patients.

**Supplementary Information:**

The online version contains supplementary material available at 10.1186/s12964-024-01729-0.

## Introduction

Acute myeloid leukemia (AML) is a heterogeneous malignant disease characterized by the abnormal proliferation of myeloid precursor cells, which leads to the impaired differentiation of normal cells and bone marrow failure, with a poor prognosis. Notably, disease relapse and drug resistance continue to be the most prominent causes of patient mortality [1]. FMS-like tyrosine kinase 3 (FLT3) is the most common mutation in AML, and FLT3-activating mutations account for approximately 30% of newly diagnosed AML cases [2, 3]. There are two types of FLT3 mutations, of which FLT3-ITD is the most common type, accounting for about 25% of all AML patients and associated with poor prognosis. However, the impact of mutations in the FLT3 tyrosine kinase domain (FLT3-TKD), which account for about 7–10% of all patients, on the prognosis of AML patients is controversial [4]. FLT3 is a member of the type III receptor tyrosine family, and wild-type FLT3 (FLT3-WT) maintains a monomeric structure when inactive; binding to its ligand (FL) induces its dimerization, which activates the downstream PI3K/AKT and MAPK pathways [5]. The ITD or TKD mutations in FLT3 lead to the constitutive activation of the kinase in a non-ligand-dependent manner, and self-dimerization can occur to activate the MAPK, PI3K/AKT, and STAT5 signaling pathways, resulting in increased cell proliferation and impaired apoptosis [6, 7]. Several tyrosine kinase inhibitors (TKIs), including first-generation FLT3 inhibitors such as tandutinib, sunitinib, midostaurin, lestaurtinib and sorafenib, have been developed as valuable therapeutic agents for prolonging overall survival in AML patients [8–12]. Due to their poor efficacy and selectivity, the research and development of second-generation FLT3 inhibitors, such as gilteritinib, quizartinib and crenolanib, is ongoing [13–15]. FLT3 inhibitors are also subdivided into type I and type II. Type I FLT3 inhibitors such as gilteritinib bind the FLT3 receptor in the active conformation and are active against ITD and TKD mutations. But type II FLT3 inhibitors such as quizartinib bind the FLT3 receptor in the inactive conformation at a region adjacent to the ATP-binding domain. As a result of the binding mode, type II FLT3 inhibitors prevent activity of ITD mutations but do not target TKD mutations [16]. Among the various FLT3 inhibitors, midostaurin plus chemotherapy was approved by the FDA in 2017 for the treatment of adult patients with newly diagnosed FLT3-mutated AML and the FDA approved quizartinib plus chemotherapy to treat newly diagnosed AML patients with FLT3-ITD mutations last year [17, 18]. To date, only gilteritinib has been approved globally as a single agent for patients with relapsed/refractory FLT3-mutated AML [19], and it has demonstrated significant clinical efficacy but has been limited by the emergence of drug resistance. Notably, only temporary remission can be achieved due to the emergence of drug resistance [4, 20]. FLT3 TKI resistance can be categorized as primary or secondary, and secondary resistance involves acquired on-target TKD mutations and off-target mutations of other genes [21]. The acquisition of FLT3-TKD mutations at D835, Y842, F691 or other sites is the most common mechanism of resistance against type II FLT3 inhibitors that bind inactive FLT3. However, the “gatekeeper” mutation (F691L) is a common and persistent resistance mechanism that confers resistance to currently used FLT3 inhibitors [22]. Therefore, there is an urgent need to develop new kinase inhibitors that can overcome drug resistance.

Ningetinib is a multikinase inhibitor that inhibits the phosphorylation of c-MET, VEGFR and AXL; blocks downstream signaling pathways; and exerts antitumor proliferative effects on a variety of solid tumor models, including lung, renal, breast, and bladder cancer models. Ningetinib has good pharmacokinetics and safety and is currently in phase I clinical trials for solid tumors, but no studies have reported the use of ningetinib for the treatment of AML [23, 24]. Our study identified ningetinib as a FLT3 inhibitor and the molecular docking suggested that ningetinib seemed to be a type II FLT3 inhibitor. Ningetinib exhibited good therapeutic effects on FLT3 mutant AML models in vitro, and showed significant antitumor effects in MOLM13-, Ba/F3-FLT3-ITD-, Ba/F3-FLT3-ITD-F691L- and MOLM13-FLT3-ITD-F691L-driven leukemia mouse models, with results superior to existing clinical drugs gilteritinib and quizartinib. Thus, ningetinib is a potent FLT3 TKI that can overcome secondary resistance, especially the gatekeeper mutation F691L, and may serve as an optional agent for AML treatment, either as a single agent or in combination with conventional chemotherapy.

## Materials and methods

### Patient sample preparation

Bone marrow (BM) samples were collected from AML patients (patient details are provided in Supplementary Table S1). BM mononuclear cells and peripheral blood mononuclear cells (PBMCs) from healthy donors were separated with Lymphoprep reagent (Stemcell Technologies) using density gradient centrifugation and cultured in RPMI-1640 supplemented with 20% FBS. Written informed consent was obtained from all patients and healthy donors in accordance with the Declaration of Helsinki, and all procedures were approved by the Institutional Review Board of Guangzhou First People’s Hospital, School of Medicine, South China University of Technology.

### Cell culture and cell lines

Human leukemia cell lines (MV4-11, MOLM-13, K562, HL-60, OCI-AML2, OCI-AML3, U937 and THP-1) were cultured with RPMI-1640 (Gibco) supplemented with 10% FBS (Gemini) and 1% penicillin‒streptomycin and then cultured in a humidified atmosphere at 37 °C with 5% CO_2_. Ba/F3 cells expressing FLT3-ITD, FLT3-ITD-F691L, FLT3-ITD-D835Y, FLT3-ITD-D835V and FLT3-ITD-Y842C were generated by retroviral infection as previously described [14]. MOLM13 cells expressing FLT3-ITD-F691L were generated by lentiviral infection as previously described [14] .

### Chemicals and reagents

Ningetinib, gilteritinib and quizartinib were purchased from TargetMol (Boston, USA). In the in vitro experiments, all the drugs were dissolved in DMSO to 10 mM, stored at -20 °C, and used after dilution, with a final DMSO concentration of < 0.1%. For in vivo animal experiments, gilteritinib and ningetinib were dissolved in a solution containing 5% DMSO, 35% PEG300, 10% Tween 80 and 50% sterile water, and quizartinib was suspended in a 0.5% methylcellulose solution.

### Cell viability assay

Cells (3 × 10^3^/well/100 µl) were seeded into 96-well plates and treated with the indicated concentrations of the corresponding drugs in triplicate. After 48 h of treatment, cell proliferation was assessed using a CellTiter-Glo^®^ 2.0 Cell Viability Assay (Promega). Luminescence was measured with a VICTOR Nivo instrument (Revvity). The mean viability (luminescence) at concentration 0 of triplicate was normalized as 100% and the cell viability at different concentrations was normalized to the percentage of the mean viability at concentration 0. Finally, the concentrations of the data were transformed by GraphPad Prism to generate dose–response curves and then the transformed data was processed by “Nonlin fit” analysis in GraphPad Prism to calculate the 50% inhibitory concentration (IC50).

### Cell apoptosis and cell cycle assays

MV4-11, and MOLM13 cells (2.0 × 10^5^/ml) were seeded in 6-well plates at which DMSO or different concentrations of ningetinib were added, and then, the cells were incubated for 24–48 h. To assess apoptosis, cells were stained using an Annexin V/propidium iodide (PI) Apoptosis Detection Kit (Invitrogen) and analyzed by flow cytometry (BD LSRFortessa). For the cell cycle analysis, cells were harvested after 24 h, washed once with cold PBS and then fixed in 70% ethanol at -20 °C overnight. Then, the cells were stained with propidium iodide (Sigma–Aldrich), and flow cytometry was performed to detect the DNA content. The results were analyzed using FlowJo software.

### Western blot analysis

Cells were washed with PBS and lysed with 2x sodium dodecyl sulfate (SDS) sample loading buffer (Sigma‒Aldrich) supplemented with protease inhibitors (TargetMol). The protein samples were then subjected to sodium lauryl sulfate polyacrylamide electrophoresis, and the separated proteins were transferred to a nitrocellulose membrane. The following antibodies were used: anti-p-FLT3 (#3464, CST), anti-FLT3 (#3462, CST), anti-p-STAT5 (#9351, CST), anti-STAT5 (#9363, CST), anti-ERK (#4695, CST), anti-p-ERK (#4370, CST), anti-AKT (#9272, CST), anti-p-AKT (#4060, CST), anti-tubulin-HRP (#HRP-66,031, Proteintech), anti-PARP1 (#13371-1-AP, Proteintech) and anti-caspase8 (#13423-1-AP, Proteintech). Images of the western blots were taken using ChemiDoc MP (Bio-Rad).

### Molecular docking

The structure of ningetinib was downloaded from the PubChem database (https://pubchem.ncbi.nlm.nih.gov/). The 3D structure was prepared, and the coordinates were determined based on the OPLS_4 force field by the LigPrep module in Schrödinger. All possible stereoisomers and associated protonation states were determined using the Epik module. Then, the crystal structure of FLT3 (6JQR) was downloaded from the PDB database, and protein preparation was performed using the Protein Preparation Wizard module in Schrödinger. The protein was prepared by assigning bond levels, hydrogenating, replacing missing side chains, removing water molecules and cofactors, optimizing the hydrogen bonding network, and finally using OPLS_4 force field for protein energy minimization. Molecular docking was performed using the Glide module in Schrödinger. Proto-crystallized ligands were used as binding pockets, with the outer box set to be similar in size to the proto-crystallized ligand and the inner box set to 10 Å. Docking was carried out using standard precision (SP docking), and the GlideScore built into Schrödinger was used as the scoring function. The lower the score was, the lower the binding free energy between the compound and the protein, and the greater the binding stability.

### Cellular thermal shift assay

The cellular thermal shift assay (CETSA) was performed as previously described [25]. Briefly, 1 × 10^7^ BaF3- FLT3-ITD cells in 10-cm dishes were incubated with ningetinib (10 µM) or DMSO for 60 min, and then, the cells were collected, washed, resuspended in PBS supplemented with protease inhibitors (TargetMol) and evenly divided into 6 portions for heat treatment at 37–54 °C for 3 min. Proteins were extracted using liquid nitrogen and quantified by immunoblotting. The protein bands were quantified by Image Lab, and the data were analyzed with GraphPad Prism.

### In vivo efficacy studies

All animal studies were approved by the Animal Ethics Committee of South China University of Technology. This study complied with all relevant ethical regulations regarding animal research. In Ba/F3 model, 5 × 10^5^ FLT3-ITD or FLT3-ITD-F691L cells were injected via the tail vein into 8-week-old female BALB/c mice. The mice were randomized into 4 groups (10 mice each) and were given continuous administration of vehicle, gilteritinib (30 mg/kg), quizartinib (10 mg/kg) or ningetinib (30 mg/kg) from day 2 until the first mouse in the vehicle group died. To assess leukemia burden, peripheral blood (PB) was collected, and three mice per group were sacrificed to collect bone marrow (BM) and spleen (SP) cells for flow cytometry. In the flow cytometry analysis, leukemic cells were defined as GFP-positive cells. Tissue morphology was visualized using hematoxylin and eosin (H&E) staining. In the MOLM13 or MOLM13-FLT3-ITD-F691L tumor xenograft model, five-week-old female NSG mice (Shanghai Model Organisms Center) were intravenously injected with 1 × 10^7^ MOLM13 or MOLM13-FLT3-ITD-F691L cells. 6 days after cell inoculation, gilteritinib (30 mg/kg), quizartinib (10 mg/ kg), ningetinib (30 mg/kg) or vehicle was dosed daily by oral gavage for 14 days. 3 mice of each group were sacrificed on day 22 and the percentage of human CD45 positive cells in bone marrow (BM) and spleen (SP) was detected by flow cytometry.

### Statistical analysis

GraphPad Prism 9.5 software was used to perform the statistical analyses. Differences between groups were analyzed utilizing paired or unpaired 2-tailed Student’s t tests (*P < 0.05; **P < 0.01; ***P < 0.001, **** *P* < 0.0001). Kaplan‒Meier survival curves and log-rank tests were used to estimate survival.

## Results

### Ningetinib significantly inhibits the activities of FLT3-ITD AML cells

**Fig. 1:**
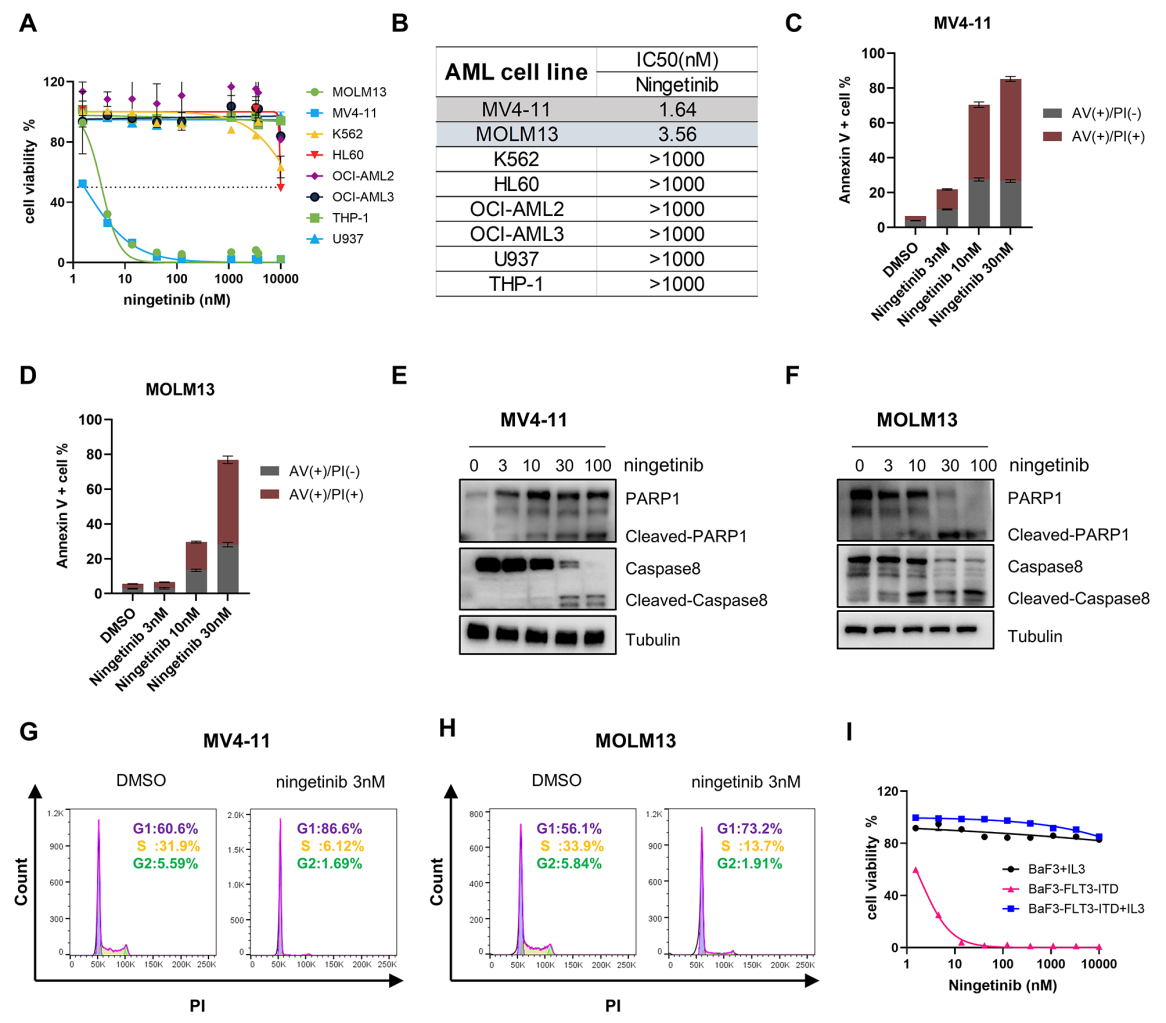
Ningetinib significantly inhibits the activities of FLT3-ITD AML cells. **A.** Dose‒response curves for the FLT3-WT and FLT3-ITD (MV4-11 and MOLM13) AML cell lines after treatment with ningetinib for 48 h. The mean viability of triplicate at concentration 0 was normalized as 100% as control. The data are representative of three experiments. **B.** IC_50_ values for ningetinib for the FLT3-WT (K562, HL60, OCI-AML2, OCI-AML3, U937, and THP-1) and FLT3-ITD (MV4-11 and MOLM13) AML cell lines. **C, D.** Proportion of apoptotic cells among the MV4-11 and MOLM13 cells treated with different concentrations of ningetinib for 48 h. **E, F.** Expression of PARP1, caspase 8, cleaved PARP1 and cleaved caspase 8 in MV4-11 and MOLM13 cells as analyzed by western blot after 48 h of treatment with different concentrations of ningetinib. **G, H.** Percentage of cells in different cell cycle phases detected by flow cytometry after treating cells with DMSO or 3 nM ningetinib for 24 h. **I.** Dose‒response curves for Ba/F3 and Ba/F3-FLT3-ITD cells treated with ningetinib for 48 h. The mean viability of triplicate at concentration 0 was normalized as 100% as control. The data are representative of three experiments

To explore the therapeutic efficacy and specificity of ningetinib on AML, we performed cell proliferation assays using a variety of human leukemia cell lines. We found that ningetinib had a significant inhibitory effect on the FLT3-ITD-expressing cell lines MV4-11 and MOLM13, with IC_50_ values of 1.64 nM and 3.56 nM, respectively, and had little cytotoxic effect on the wild-type FLT3 (FLT3-WT)-expressing cell lines (K562, HL60, OCI-AML2, OCI-AML3, U937, and THP-1) (Fig. 1A, B). Subsequently, after treatment with ningetinib for 48 h, we found a dose-dependent increase in the proportion of Annexin V-positive cells (MV4-11 and MOLM13) (Fig. 1C, D and Fig. S1A) and the concurrent activation of the apoptotic protein PARP1 and cleavage of caspase 8 (Fig. 1E, F). Moreover, a cell cycle assay revealed that 24 h of ningetinib treatment resulted in cell cycle arrest in the G1 phase in a dose-dependent manner (Fig. 1G, H). We also evaluated the survival of FLT3-ITD-dependent Ba/F3-FLT3-ITD cells to determine the specificity of ningetinib for FLT3 mutations. Ningetinib effectively inhibited the growth of Ba/F3-FLT3-ITD cells, but this inhibitory effect was reversed by coculture with IL-3. And ningetinib treatment has little effect on parental Ba/F3 cells, suggesting that the selective and potent activity of ningetinib is derived from the inhibition of FLT3-ITD (Fig. 1I). Previous studies have shown that plasma protein binding is a major factor limiting the clinical efficacy of FLT3 inhibitors like midostaurin [26]. We compared the IC50 values of ningetinib and quizartinib on MV4-11 and MOLM13 cells cultured in the plasma of 100% AML patients. Addition of human plasma increased the IC50 values for ningetinib and quizartinib. However, the IC50 values of quizartinib (59.02 nM for MV4-11 and 101.3 nM for MOLM13) were significantly higher than those of ningetinib (3.37 nM for MV4-11 and 25.67 nM for MOLM13) (Fig. S1B). These results suggest that ningetinib specifically and effectively inhibits the growth of FLT3-ITD mutant cells in vitro.

### Ningetinib inhibits the FLT3 signaling pathway and exhibits significant antitumor effects in vivo

**Fig. 2:**
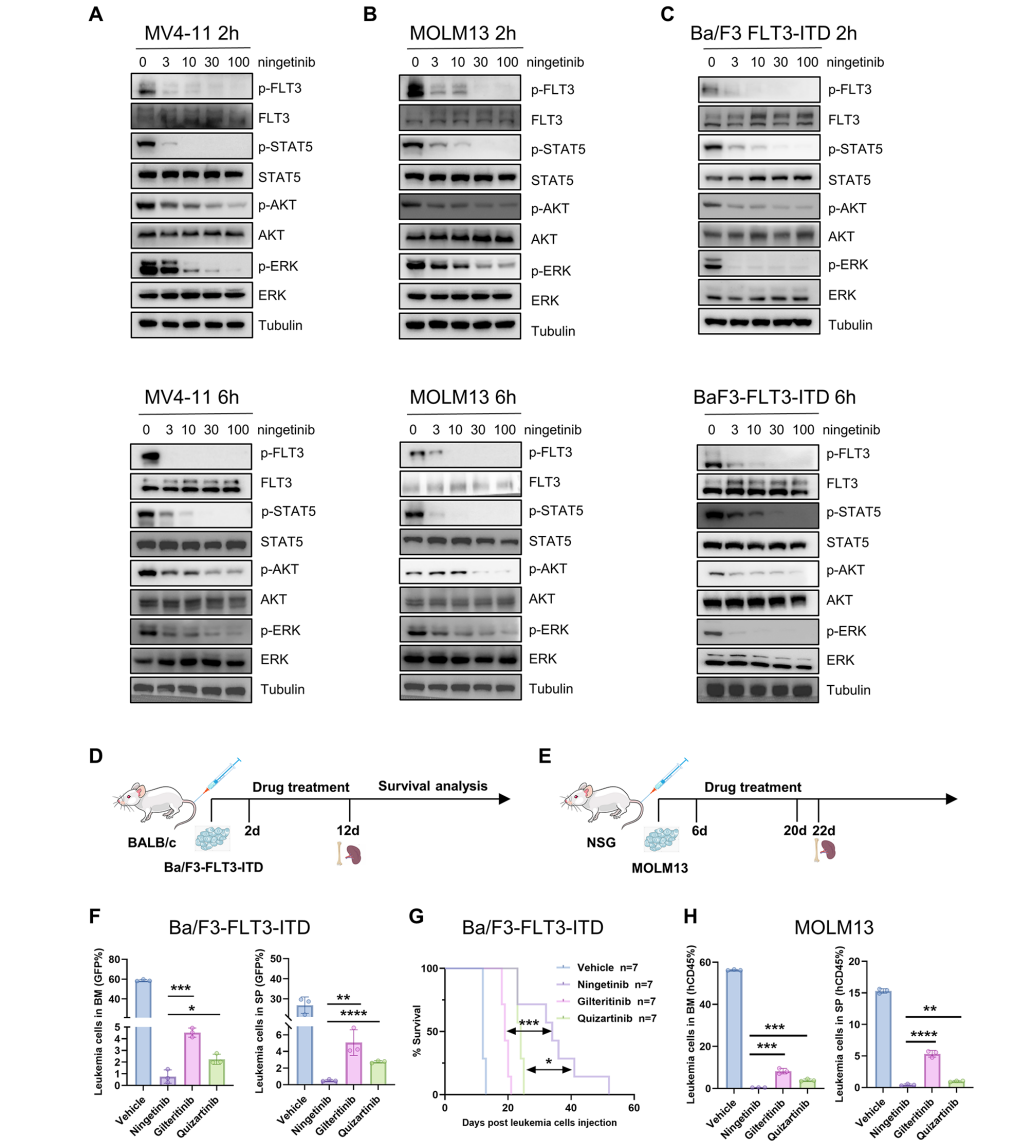
Ningetinib inhibits FLT3 phosphorylation and exhibits antitumor activity against FLT3-ITD mutation in vivo. **A, B, C.** Western blot analysis of p-FLT3, FLT3, p-STAT5, STAT5, p-AKT, AKT, p-ERK and ERK in the MV4-11 and MOLM13 cells after treatment with ningetinib at the indicated doses for 2 h or 6 h. **D.** Schematic representation of the leukemic mouse model induced by Ba/F3-FLT3-ITD cells. **E.** Schematic representation of xenograft experiments using human MOLM13 cells. **F.** Proportion of GFP-positive cells in the BM and SP of mice, measured by flow cytometry on Day 12 (*n* = 3 mice per group). **G.** The survival curves of BaF3-FLT3-ITD-diseased mice treated with the vehicle (*n* = 7), ningetinib (*n* = 7), gilteritinib (*n* = 7) or quizartinib (*n* = 7). **H.** The percentage of human CD45 positive cells in BM and SP of MOLM13-diseased NSG mice detected by flow cytometry on day 22 (*n* = 3 mice per group). Error bars indicate mean ± standard error, *P < 0.05, **P < 0.01, ***P < 0.001, ****P < 0.0001

We treated MV4-11 and MOLM13 cells with different concentrations of ningetinib for 2h or 6h and then assessed the phosphorylation of FLT3 and its downstream target proteins STAT5, AKT and ERK by western blot analysis. Ningetinib inhibited the phosphorylation of FLT3 and its downstream target proteins STAT5, AKT and ERK in a dose- and time-dependent manner, serving as the mechanism by which ningetinib has significant antileukemic effects on FLT-ITD-positive AML cells (Fig. 2A, B). We also performed the same experiment in Ba/F3-FLT3-ITD cells (Fig. 2C). Then, we constructed mouse models with FLT3-ITD mutation to evaluate the efficacy of ningetinib in vivo (Fig. 2D, E). In Ba/F3-FLT3-ITD mouse model, GFP-positive Ba/F3-FLT3-ITD cells were injected into BALB/c mice, and the drug was administered starting on Day 2 (Fig. 2D). The proportion of GFP-positive cells in the PB of the mice was assessed on Day 10. In all treatment groups, the leukemic burden in the PB was significantly lower than that in the PB of mice in the vehicle group, with the greatest effect in the ningetinib group (27% in the vehicle group, 3% in the gilteritinib group, 0.55% in the quizartinib group, and 0.27% in the ningetinib group) (Fig. S2A, B). Three mice were randomly selected from each group, and the leukemic burden in the BM and SP were analyzed on Day 10. Compared with those in the vehicle group, the proportions of leukemia cells in the BM and SP in the treatment group were markedly lower, and compared with the other 3 groups, the proportion of leukemia cells in the ningetinib group was lower (Fig. 2F and Fig. S2A). There was no significant weight loss or other serious toxic effects in any of the groups of mice during the dosing period (Fig. S2C). Ningetinib significantly prolonged the median survival of mice from 12 days in the vehicle group to 34 days, while compared with the vehicle, gilteritinib only extended the median survival by 7 days (Fig. 2G). We further performed allogeneic bone marrow transplantation experiments on NSG mice using MOLM13 cells. The mice were administrated vehicle, ningetinib (30 mg/kg), gilteritinib (30 mg/kg) or quizartinib (10 mg/kg) daily for 14 consecutive days (Fig. 2E). Compared with quizartinib and gilteritinib, ningetinib also decreased the percentage of human CD45 positive cells in BM and SP to a greater extent in MOLM13 mouse model (Fig. 2H and Fig. S3A). And no obvious weight loss or any other signs of toxicity were observed among the groups for a total of 23 days during and after ningetinib treatment (Fig. S3B). Thus, ningetinib exhibited favorable antileukemic effects both in vivo and in vitro.

### Ningetinib binds to FLT3 directly with high affinity

**Fig. 3:**
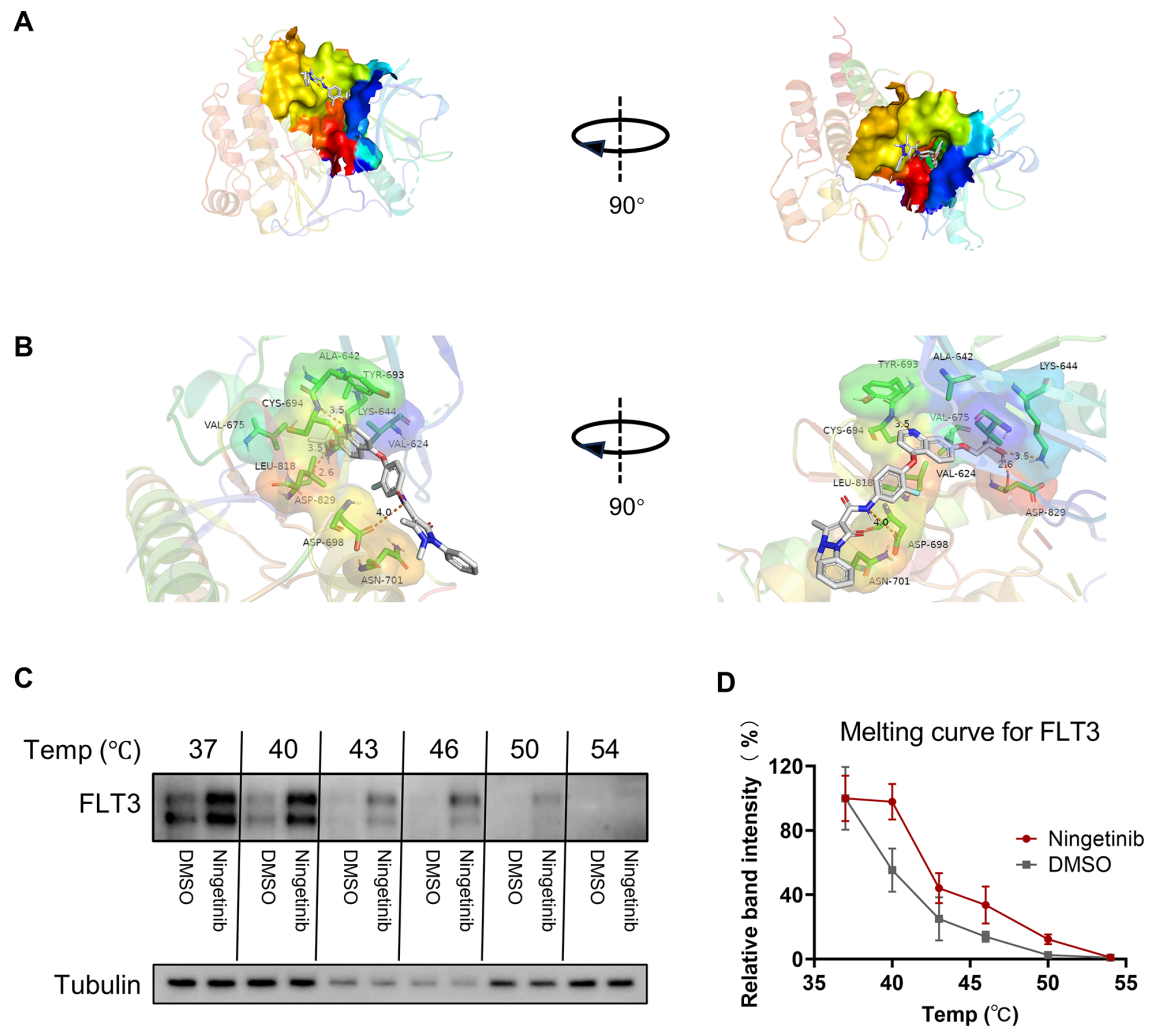
The binding of ningetinib to FLT3. **A.** Overview of the docking results for ningetinib and FLT3 (Protein Data Bank: 6JQR) from two orthogonal views. **B.** Detailed docking sites for the binding of ningetinib and FLT3. Proteins are shown as cartoons, active pockets are shown as surfaces, green sticks are interacting amino acid residues, white sticks are small molecules, and orange dashed lines are hydrogen bonds. **C.** Ba/F3-FLT3-ITD cells were treated with ningetinib (10 µM) or DMSO for 1 h. The temperature ranged from 37 to 55 °C for testing. **D.** Proteins were quantified using ImageJ, and melting curves for FLT3 were plotted. The data are presented as means ± standard errors from three independent experiments

To assess the interaction of ningetinib with FLT3, we performed virtual molecular docking of ningetinib to the ATP of FLT3. The predicted minimum binding capacity of ningetinib for the FLT3 protein was − 9.269. The docking results showed that ningetinib can occupy the ATP pocket of FLT3 well and form 1 key hydrogen bond with the amino acid residue Cys-694 in the hinge region, with an interaction distance of 3.5 Å. In addition, hydrogen bonding interactions were formed with Lys-644, Asp-829 and Asp-698 at distances of 3.5 Å, 2.6 Å and 4.0 Å, respectively; furthermore, Asn-701, Leu-818, Val-624, Val-675, Tyr-693 and Ala-642 formed hydrophobic interactions, facilitating strong binding between ningetinib and FLT3 (Fig. 3A, B). Compared with those of DMSO-treated samples, the dissolution profiles of ningetinib-treated samples exhibited a significant thermal shift, with the FLT3 protein almost completely disappearing from the DMSO-treated samples at 46 °C, whereas ningetinib was still detectable at 50 °C (Fig. 3C, D), confirming the direct interaction between ningetinib and FLT3.

### Ningetinib overcomes FLT3 inhibitor resistance caused by secondary mutations

**Fig. 4:**
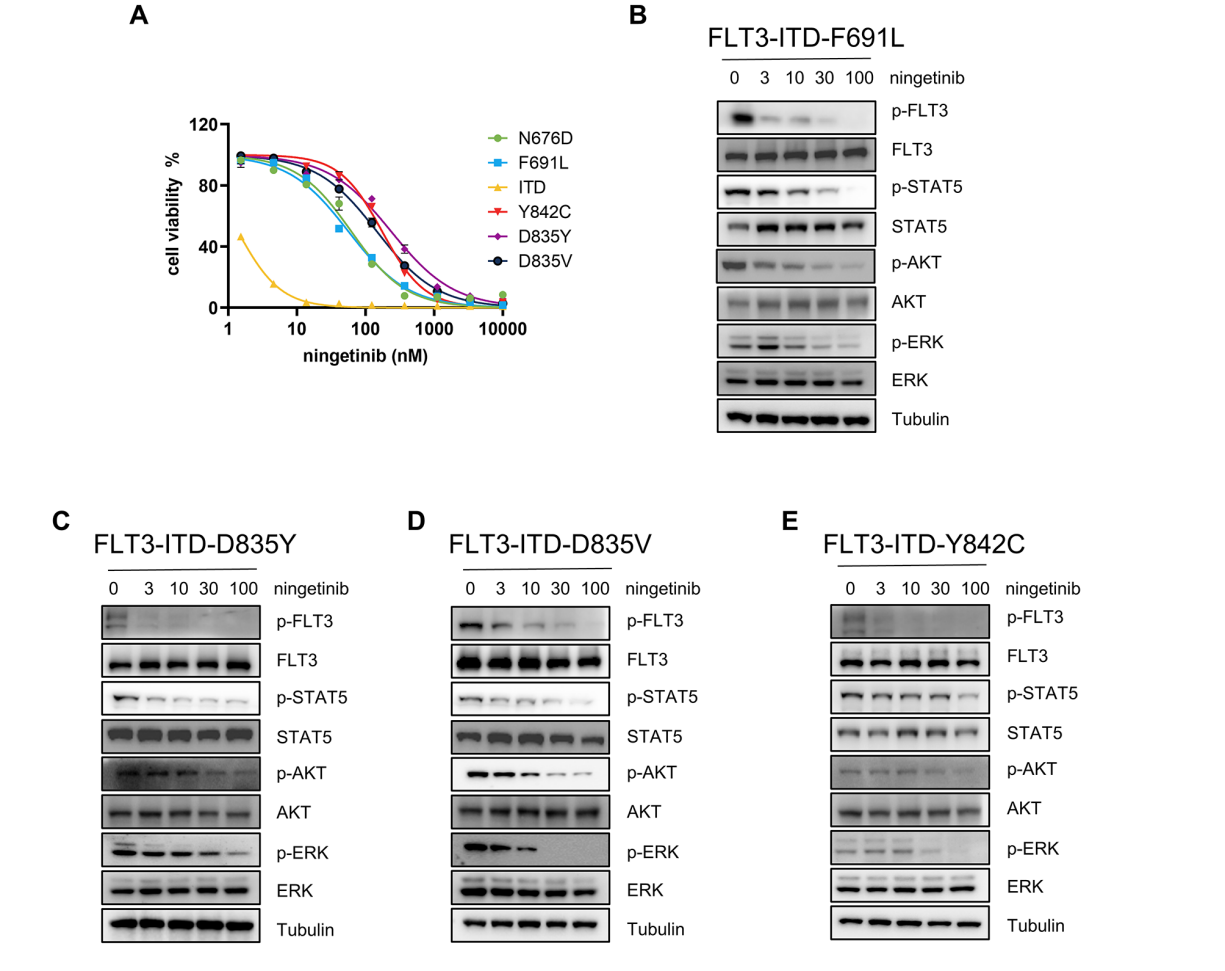
Ningetinib inhibits secondary resistant TKD mutations in vitro. **A.** Dose‒response curve for cells with secondary mutations (FLT3-ITD-D835Y/D835V/Y842C/N676D/F691L) after treatment with ningetinib for 48 h. The mean viability of triplicate at concentration 0 was normalized as 100% as control. Error bars indicate the mean ± standard error, *n* = 3 independent experiments. **B, C, D, E.** Western blot analysis of p-FLT3, FLT3, p-STAT5, STAT5, p-AKT, AKT, p-ERK and ERK expression in the FLT3-ITD-D835Y/D835V/Y842C/F691L cells treated with ningetinib for 6 h. Tubulin was used as a loading control

We generated Ba/F3 cells with FLT3-ITD-D835Y/D835V/Y842C/N676D/F691L mutations and performed cell proliferation assays. Ningetinib had an inhibitory effect on all of these cells, especially the F691L mutation, with a significantly stronger effect than quizartinib (IC_50_ 56.1nM vs. 484.3nM) (Fig. 4A and Fig. S4A). Western blot analysis of FLT3-ITD-D835Y/D835V/Y842C/F691L cells after treatment with different concentrations of ningetinib for 6 h revealed that ningetinib inhibited the phosphorylation of FLT3 and its downstream target proteins STAT5, AKT, and ERK (Fig. 4B-E).

### Ningetinib overcomes secondary resistance caused by FLT3-ITD-F691L mutations in vivo

**Fig. 5:**
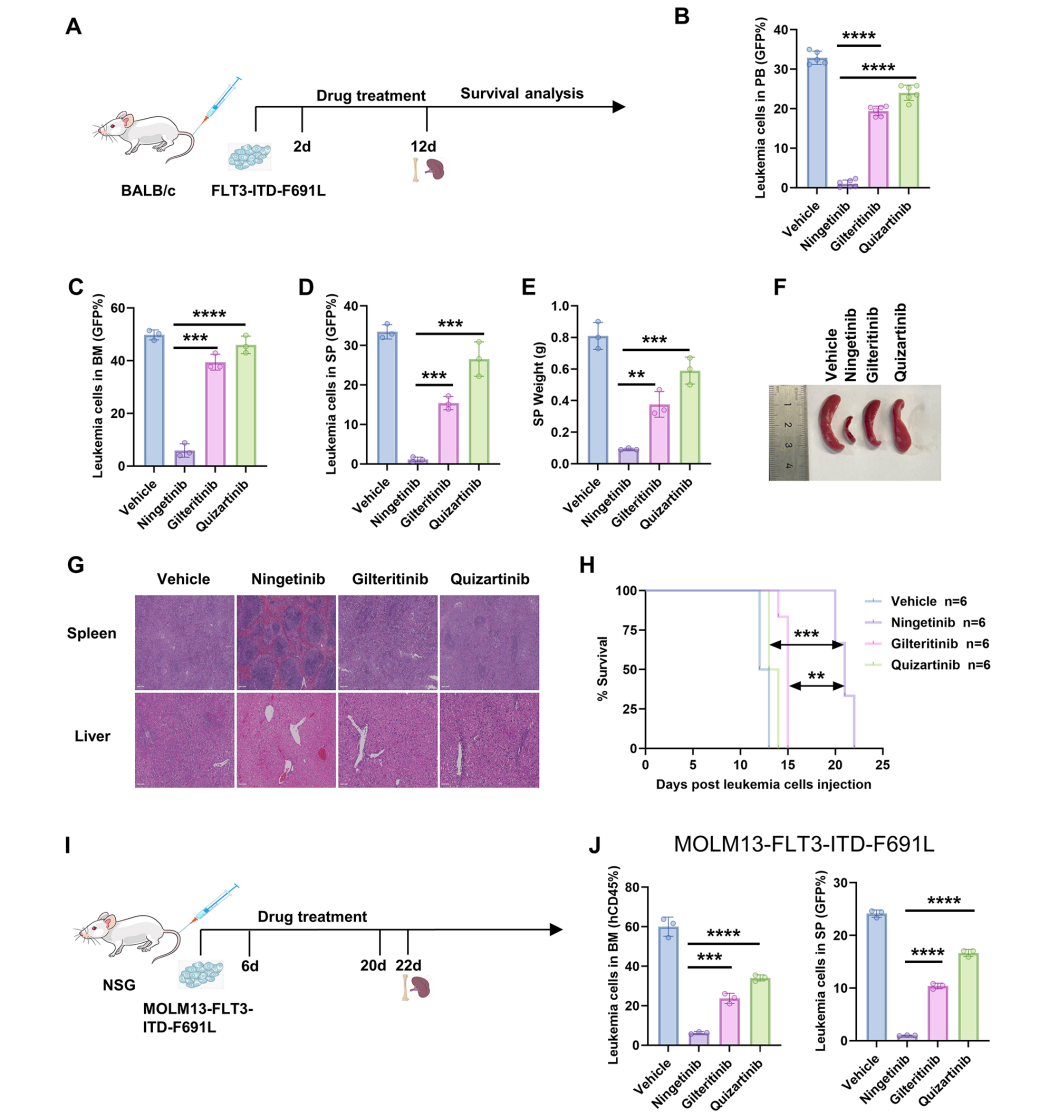
Ningetinib exerts efficient inhibitory effects on FLT3-ITD-F691L mutations in vivo. **A.** Schematic representation of the mouse model of leukemia induced by Ba/F3-FLT3-ITD-F691L cells. **B.** Percentage of GFP-positive cells in PB of BALB/c mice, measured by flow cytometry on Day 10 (*n* = 6 mice per group). **C, D.** Proportions of GFP-positive cells in BM and SP of BALB/c mice, examined by euthanizing 3 mice per group on Day 12. **E.** Weights of the spleens of the sacrificed mice in C (*n* = 3 per group). **F.** Images of the spleens of sacrificed mice in E. **G** Images of the H&E-stained mouse livers and spleens. **H.** Survival curves for BaF3-FLT3-ITD-F691L diseased mice treated with the vehicle (*n* = 6), ningetinib (*n* = 6), gilteritinib (*n* = 6) or quizartinib (*n* = 6). **I.** Schematic representation of xenograft experiments using human MOLM13-FLT3-ITD-F691L cells. **J.** The percentage of human CD45 positive cells in BM and SP of MOLM13-FLT3-ITD-F691L diseased NSG mice detected by flow cytometry on day 22 (*n* = 3 mice per group). Data are presented as the mean ± SD. *P < 0.05, **P < 0.01, ***P < 0.001, ****P < 0.0001

First, we used BALB/c mice to construct FLT3-ITD-F691L leukemia model to evaluate the efficacy of ningetinib in vivo (Fig. 5A). We injected GFP-positive Ba/F3-FLT3-ITD-F691L cells into mice, which were then randomly divided into four groups, including a vehicle group and groups treated with ningetinib, gilteritinib or quizartinib starting on Day 2. Subsequently, the proportion of GFP-positive cells in the PB, BM, and SP of mice was examined by flow cytometry. Compared with mice treated with gilteritinib or quizartinib, mice treated with ningetinib had a lower leukemic burden (Fig. 5B-D and Fig. S5A). And compared with those in mice treated with gilteritinib or quizartinib, the sizes of spleens in mice treated with ningetinib were significantly reduced (Fig. 5E, F) and there was no significant tissue or structural disruption in the livers and spleens (H&E staining) (Fig. 5G). Ningetinib significantly prolonged the median survival of mice, outperforming gilteritinib and quizartinib (21 days vs. 15 and 13 days) (Fig. 5H). To further verify the in vivo efficacy of ningetinib against FLT3-F691L secondary mutations, we performed allogeneic bone marrow transplantation experiments using MOLM13-FLT3-ITD-F691L cells. The mice were administrated vehicle, ningetinib (30 mg/kg), gilteritinib (30 mg/kg) or quizartinib (10 mg/kg) daily for 14 consecutive days (Fig. 5I).

In MOLM13-FLT3-ITD-F691L mouse model, ningetinib significantly decreased the percentage of human CD45 positive cells in BM (6.87%) and SP (1.03%) while quizartinib (35.51% in BM, 17.06% in SP) and gilteritinib (24.21% in BM, 10.55% in SP) exhibited much weaker efficacy (Fig. 5J and Fig. S5B). These results suggest that ningetinib can overcome clinical resistance caused by secondary F691L mutations both in vitro and in vivo and could be a potential drug for the treatment of AML.

### Ningetinib demonstrates therapeutic potential for patients with FLT3-ITD mutations

**Fig. 6:**
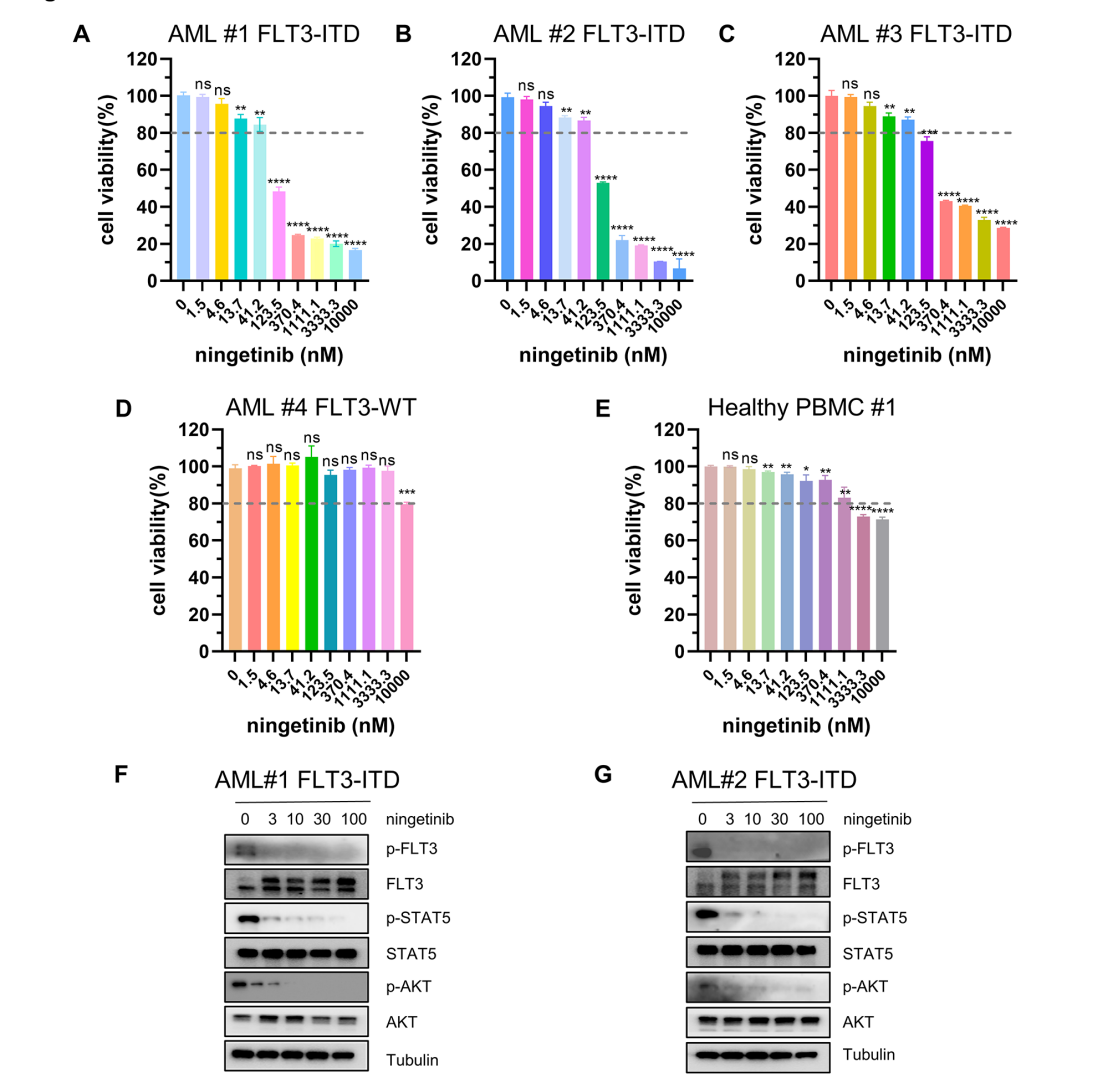
Ningetinib exhibits therapeutic potential in primary cells derived from patients with FLT3-ITD mutations. **A, B, C, D.** Cell viability values for primary BM cells from AML patients with FLT-ITD or FLT3-WT after treatment with different concentrations of ningetinib. The data are representative of three experiments. **E.** Cell viability values for PBMCs from healthy donors after treatment with different concentrations of ningetinib. **F, G.** Primary cells from FLT3-ITD-positive AML patients were cultured with ningetinib for 12 h. Proteins were extracted, and the expression of p-FLT3, FLT3, p-STAT5, STAT5, p-AKT, AKT, p-ERK and ERK was assessed by western blot analysis. Data are presented as the mean ± SD. *P < 0.05, **P < 0.01, ***P < 0.001, ****P < 0.0001

To verify the therapeutic potential of ningetinib in clinical, we collected bone marrow cells from 2 FLT3-WT and 3 FLT3-ITD-mutant AML patients for cell proliferation assays. Ningetinib had a significant inhibitory effect on the proliferation of FLT3-ITD-mutated cells over 100 nM (Fig. 6A-C, Fig. S6). In contrast, FLT3-WT primary cells were insensitive to ningetinib up to 3000 nM, confirming the selectivity of ningetinib on FLT3-ITD mutations (Fig. 6D and Fig. S6). We also tested the effect of ningetinib on PBMCs from 4 healthy individuals and found that ningetinib had little or no inhibitory effect on normal PBMCs up to 1000nM, suggesting that ningetinib treatment at the effective concentration against FLT3-ITD blasts had good safety in myelosuppression (Fig. 6E and Fig. S6).

Western blot analysis revealed that ningetinib inhibited the phosphorylation of FLT3 and downstream pathway proteins in the primary cells of two patients with FLT3-ITD mutations (Fig. 6F, G), indicating that the antileukemic effect of ningetinib on primary cells in patients is related to the inhibition of the FLT3 signaling pathway.

## Discussion

AML is a highly heterogeneous disease, and the classification of AML has gradually shifted from a morphological scheme to a scheme determined by the causative genome. FLT3 is the most common mutation type in AML. Guidelines recommend rapid molecular testing for FLT3 mutations at the time of diagnosis and the early addition of targeted agents to achieve deeper remission and prolong patient survival, resulting in widespread advances in knowledge regarding FLT3 in recent years [2, 19]. Currently, gilteritinib and midostaurin have received regulatory approval in most parts of the world and are listed as recommended drugs in guidelines [27]. Quizartinib was recently approved by the FDA for use in combination with chemotherapy in newly diagnosed FLT3-ITD-positive AML patients, given its excellent performance in a phase III clinical study [18]. Although the use of FLT3 inhibitors has led to significant improvements in clinical outcomes, there are still considerable limitations. For example, due to poor monotherapy outcomes, midostaurin can only be used in combination with conventional chemotherapy in newly diagnosed patients, and it is not indicated for elderly patients [28]; furthermore, quizartinib has been shown to have dose-dependent adverse effects, such as severe QT prolongation with ventricular arrhythmia [13]. Gilteritinib is currently the only approved monotherapy for the treatment of relapsed/refractory FLT3-ITD-AML, with a CRc rate of approximately 45-55%. Compared with salvage chemotherapy, gilteritinib as a monotherapy reduced the risk of death by 36%, with a median OS of 9.3 months vs. 5.6 months (*P* < 0.001); however, approximately one-third of patients were nonresponders, and only 37% survived beyond one year [15]. Notably, drug resistance is the key reason to reduce the efficacy of FLT3 inhibitors and cause AML relapse. Secondary FLT3-TKD mutations at D835(Y/V), Y842(C) and F691(L) sites are the most common mechanism of resistance to type II FLT3 inhibitors [29]. Type I FLT3 inhibitors can overcome FLT3-TKD mutations but some treated patients also develop F691L mutations and most of the patients relapsed as a result of acquired mutation of other genes and the activation of bypass pathways [21, 30]. In a study of gilteritinib monotherapy, F691L mutation were detected in 5 of 41 patients and RAS/MAPK pathway mutations were detected in 15 of 41 patients after treatment [21]. These factors contribute to lower response rate and unsatisfied survival of patients. Therefore, there is an urgent need to develop novel FLT3 inhibitors, especially those that can overcome drug resistance and have low toxicity. Herein, we evaluated the efficacy of ningetinib in the treatment of FLT3-mutant AML. Interestingly, we found that ningetinib outperformed the other tested drugs in the in vivo experiments in mouse models of leukemia driven by either FLT3-ITD or FLT3-ITD-F691L. These experiments showed that the survival of ningetinib treated mice was significantly prolonged, and the results were better than clinically available drugs gilteritinib and quizartinib.

Ningetinib is a novel TKI that inhibits the MET, VEGFR and AXL signaling pathways. Our study revealed that ningetinib specifically inhibited the proliferation of FLT3-ITD-positive mutant cell lines (MOLM13 and MV4-11), blocked the activation of FLT3 and downstream signaling pathways, induced apoptosis, and blocked the cell cycle at the G1 phase. Subsequently, based on molecular docking, ningetinib was predicted to bind well to the ATP pocket of FLT3-ITD protein. The CETSA experiment confirmed that ningetinib binds to FLT3 directly. We next conducted experiments using cells (including D835Y/D835V, Y842C, F691 and N676D) and animal models of acquired secondary resistance mutations, and the results validated the efficacy of ningetinib in resistance models. Considering the predicted binding sites of ningetinib and its activity against secondary mutations, we speculate ningetinib is a type II FLT3 inhibitor. The excellent efficacy of ningetinib may be related to the good pharmacokinetics of ningetinib in vivo. In previous clinical studies, ningetinib was shown to have better pharmacokinetics than gilteritinib and quizartinib. Quizartinib (60 mg/kg, 15 days) had a C_max_ of 283 ng/ml (504 nM) and an AUC_0 − 24 h_ of 5080 ng·h/mL [31], and gilteritinib (80 mg/kg, 15 days) had a C_max_ of 396 ng/ml (716 nM)and an AUC_0 − 24 h_ of 6234 ng·h/mL [32] whereas ningetinib (60 mg/kg, 28 days) had a C_max_ of up to 2720 ng/ml (4487 nM) and an AUC_0 − 24 h_ of up to 37,000 ng·h/mL in the clinical trial of solid tumor [23, 24]. Additionally, according to our experimental results, the IC50 values of ningetinib for MV4-11 and MOLM13 cells in AML patient plasma is 3.37 nM and 25.67 nM, respectively, while that of quizartinib is 59.02 nM and 101.3 nM. It can be inferred that ningetinib could achieve effective concentration and exhibit single agent activity in AML patients, which need to be proved by phase 1 clinical trial of ningetinib in AML. Clinical studies of solid tumors have shown a favorable safety profile for ningetinib, with almost no grade 3 or higher treatment-related adverse events (TRAEs) at the recommended therapeutic dose of 60–100 mg [23].

c-MET and AXL are associated with tumor progression and a poor prognosis [33, 34], and previous studies have shown that the activation and increased expression of AXL are critical for the development of resistance to midostaurin and quizartinib [35, 36]. Among the second-generation inhibitors, gilteritinib is an AXL/FLT3 inhibitor, with literature also reporting its inhibition of NTRK and ALK [37, 38]. Quizartinib is reported to specifically target FLT3 without significant inhibition of other kinases [39]. Previous studies and our experimental results suggest that quizartinib is more effective in inhibiting FLT3 at the cellular level compared to gilteritinib [40]. However, the results of phase III clinical trial show that gilteritinib monotherapy is superior to quizartinib [15, 41]. AML is a heterogeneous disease and it has been demonstrated that individual patients bearing various subclones with different mutations [42]. Under the pressure of treatment, there are clonal evolution and dominant clonal transformation [21, 42]. We assume that muti-kinase inhibitor might be better to inhibit potential resistant pathways and eliminate multiple clonal population than single-target drugs. However, this needs to be further validated and the balance of toxicity and efficacy for muti-kinase inhibitor is important. It is also necessary to uncover the complex pathogenesis of AML more clearly so as to achieve more precise and comprehensive inhibition. Previous studies have demonstrated that ningetinib simultaneously targets c-MET, AXL and MERTK, inhibiting their phosphorylation and downstream signaling pathways [24]. Those effects may be other reasons for the excellent performance of ningetinib in the in vivo experiments. In conclusion, our study demonstrates that ningetinib has efficient antiproliferative effect on AML with FLT3-ITD mutations and deserves further investigation in clinical trials.

## Conclusion

The results of our study demonstrated that ningetinib is a potent inhibitor of mutated FLT3 and can overcome secondary resistance in AML, especially the gatekeeper mutation F691L. Given its safety and high bioavailability in solid tumor studies, it is hoped that ningetinib can be used soon as a single agent or in combination with conventional chemotherapy in patients with FLT3-mutated AML.

### Electronic supplementary material

Below is the link to the electronic supplementary material.

Supplementary Material 1

Supplementary Material 2

## Data Availability

No datasets were generated or analysed during the current study.

## Abbreviations

OS: Overall survival
TKI: Tyrosine kinase inhibitor
FLT3: FMS-like tyrosine kinase 3
ITD: Internal tandem duplication
AML: Acute myeloid leukemia
TKD: Tyrosine kinase domain
SP: Spleen
BM: Bone marrow
PB: Peripheral blood
FL: FLT3 ligand
IC50: The 50% inhibitory concentration
TRAE: Treatment-related adverse events
C_max_: Maximum plasma concentration
AUC_0 − 24 h_: Area under the concentration-time curve over 24 h
