# Supplementary material for: Ningetinib, a novel FLT3 inhibitor, overcomes secondary drug resistance in acute myeloid leukemia
Source: Cell Commun Signal. 2024 Jul 8;22:355. doi: 10.1186/s12964-024-01729-0 (PMC11229190; doi:10.1186/s12964-024-01729-0)
Supplement: Supplementary file 1 — Supplementary Material 1 [file 12964_2024_1729_MOESM1_ESM.docx]

**Supplemental Information for Manuscript “Ningetinib, a novel FLT3 inhibitor, overcomes secondary drug resistance in acute myeloid leukemia”**

**Fig.S1 Ningetinib induces apoptosis and inhibits cell viability in MV4-11 and MOLM13 cells. A.** Representative flow cytometry image of apoptosis assays. MV4-11 and MOLM13 cell lines were treated with indicated doses of ningetinib for 48 h. Apoptosis was detected by the Annexin V/PI assay. **B.** Dose‒response curves of MV4-11 and MOLM13 cells in 100% AML patient plasma treated with increasing concentrations of ningetinib or quizartinib for 48 hours. Data are representative of three experiments.

 **Fig.S2 Ningetinib exerts antileukemia activity in Ba/F3-FLT3-ITD-diseased BALB/c mice. A.** Representative flow cytometry image showing the percentage of leukemia cells that infiltrated in the PB, BM and SP of mice from the Ba/F3-FLT3-ITD-induced mouse model. **B.** Percentage of GFP-positive cells in the PB of mice, measured by flow cytometry on Day 10 (n = 6 mice per group). **C.** Body weights of Ba/F3-FLT3-ITD-diseased mice in each group during drug treatment.

**Fig. S3 Ningetinib exerts antileukemia activity in MOLM13-diseased NSG mice with good safety. A.** Representative flow cytometry image showing the percentage of leukemia cells (hCD45+) that infiltrated in the BM and SP of mice from the MOLM13 AML mouse model. **B.** Body weights of MOLM13-diseased NSG mice in each group during drug treatment.

**Fig.S4 Ningetinib is effective to inhibit secondary resistant mutations in vitro.** IC50 values of ningetinib and quizartinib for BaF3-FLT3-ITD cells with or without secondary TKD mutations. Error bars indicate mean ± standard error, 3 independent experiments.

**Fig.S5 Ningetinib inhibits the proliferation of leukemia cells with secondary F691L mutations in vivo. A.** Representative flow cytometry image showing the percentage of leukemia cells that infiltrated in the PB, BM and SP of mice from the Ba/F3-FLT3-ITD-F691L-induced mouse model. **B.** Representative flow cytometry image showing the percentage of leukemia cells (hCD45+) that infiltrated in the BM and SP of mice from the MOLM13-FLT3-ITD-F691L AML mouse model.

**Fig.S6 Ningetinib selectively inhibits the cell viability of primary samples harboring FLT3-ITD mutations with good safety.** Dose-response curves of primary AML samples harboring FLT3-ITD (AML #1, AML #2 and AML #3) and FLT3-WT (AML #4 and AML #5), and normal PBMCs after treatment with the indicated concentrations of ningetinib for 48 hours. The mean viability of triplicate at concentration 0 was normalized as 100% as control.

Supplementary Table S1: Clinical information relevant to AML patient samples.

| **Patient lD** | **Disease state** | **Age** | **Sex** | **Sample type** | **FLT3 Status** | **Other mutations** |
| --- | --- | --- | --- | --- | --- | --- |
| AML#1 | untreated | 58 | female | BM | FLT3-ITD | DNMT3A, MYC |
| AML#2 | untreated | 51 | female | BM | FLT3-ITD | NPM1 |
| AML#3 | untreated | 57 | female | BM | FLT3-ITD | CEBPA |
| AML#4 | untreated | 53 | male | BM | FLT3-WT | ASXL1, CEBPA, IDH2 |
| AML#5 | untreated | 29 | male | BM | FLT3-WT | DNMT3A, KRAS |
