# Supplementary material for: Ningetinib, a novel FLT3 inhibitor, overcomes secondary drug resistance in acute myeloid leukemia
Source: Cell Commun Signal. 2024 Jul 8;22:355. doi: 10.1186/s12964-024-01729-0 (PMC11229190; doi:10.1186/s12964-024-01729-0)

Fig.1E

MV4-11

PARP1

Caspase8

Tubulin

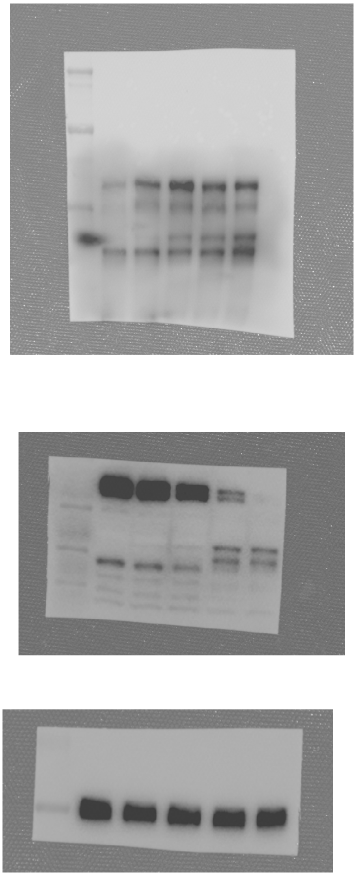

Fig.1F

MOLM13

PARP1

Caspase8

Tubulin

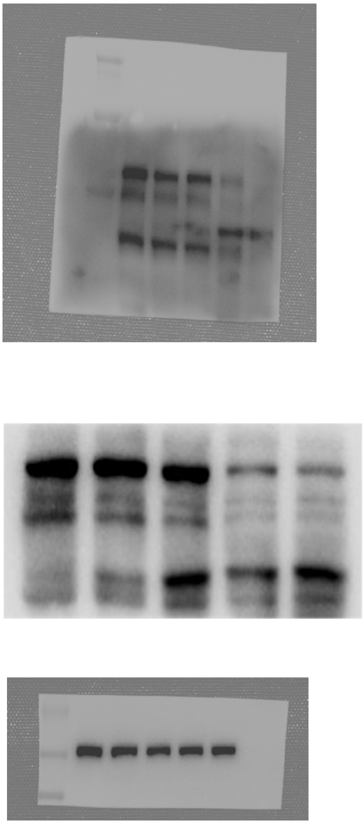

Fig.2

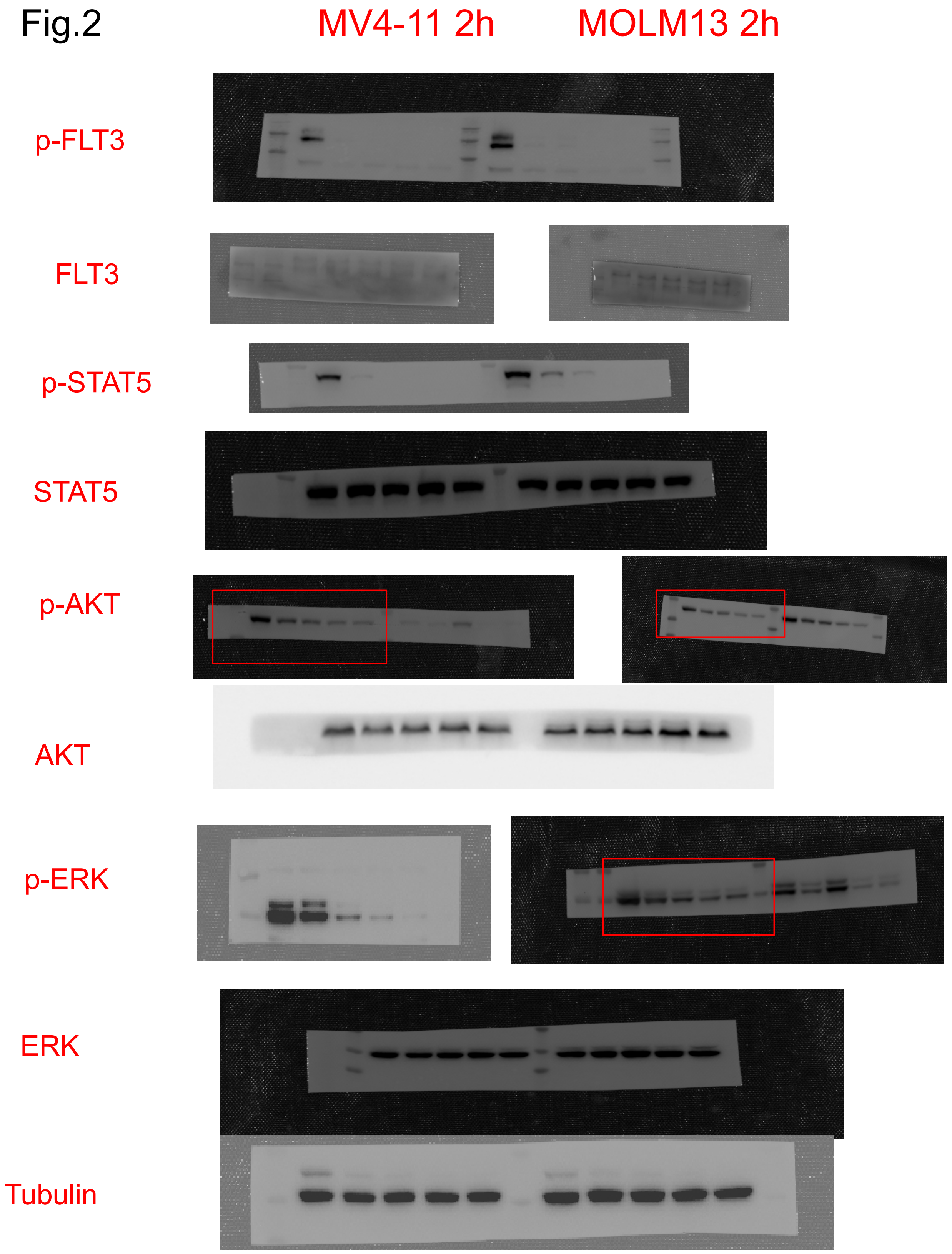
MV4-11 2h
MOLM13 2h
p-FLT3
FLT3
p-STAT5
STAT5
p-AKT
AKT
p-ERK
ERK
Tubulin

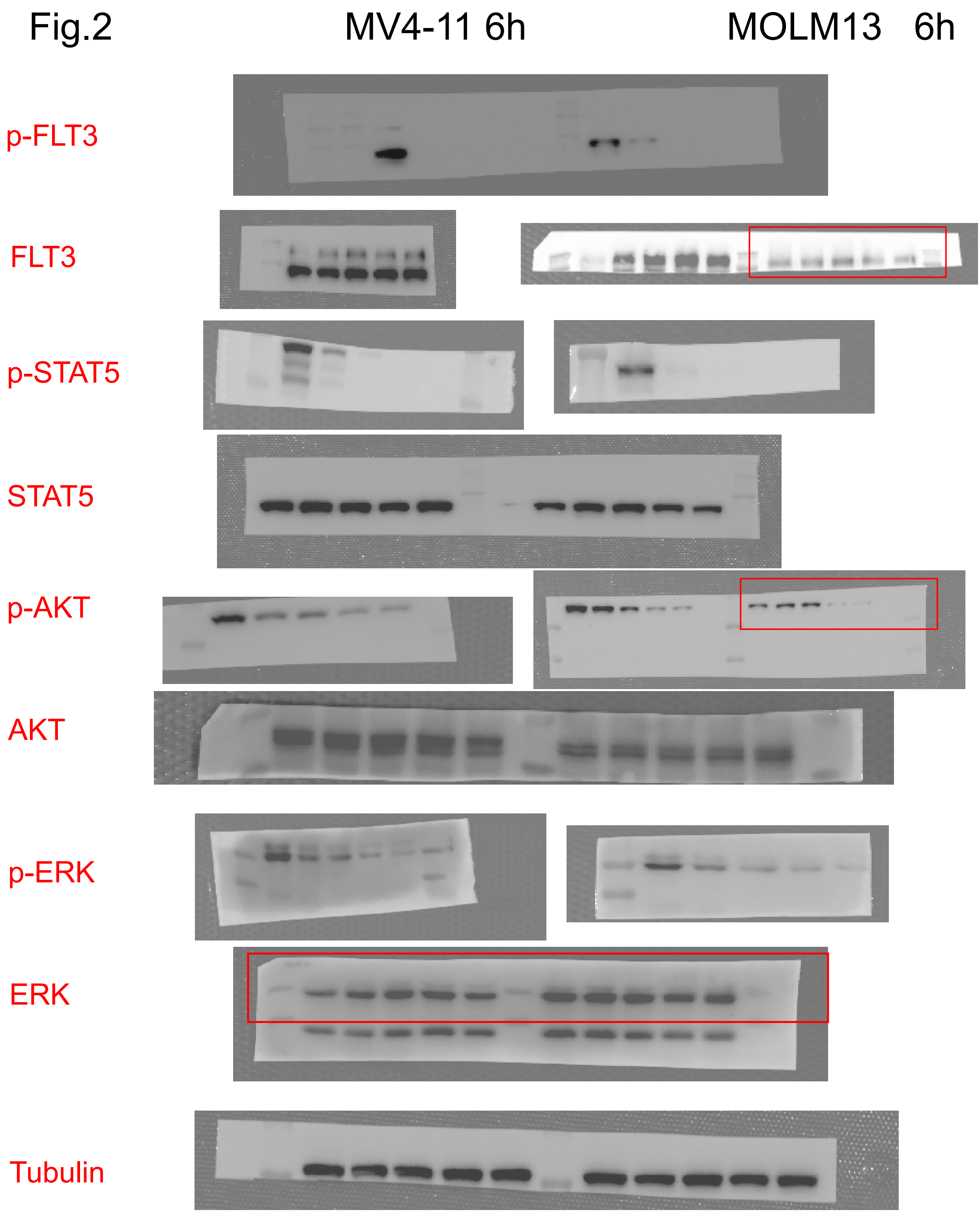
Fig.2
MV4-11 6h
MOLM13 6h
p-FLT3
FLT3
p-STAT5
STAT5
p-AKT
AKT
p-ERK
ERK
Tubulin

Fig.2 Ba/F3 FLT3-ITD 2h

Fig.4 FLT3-ITD-F691L

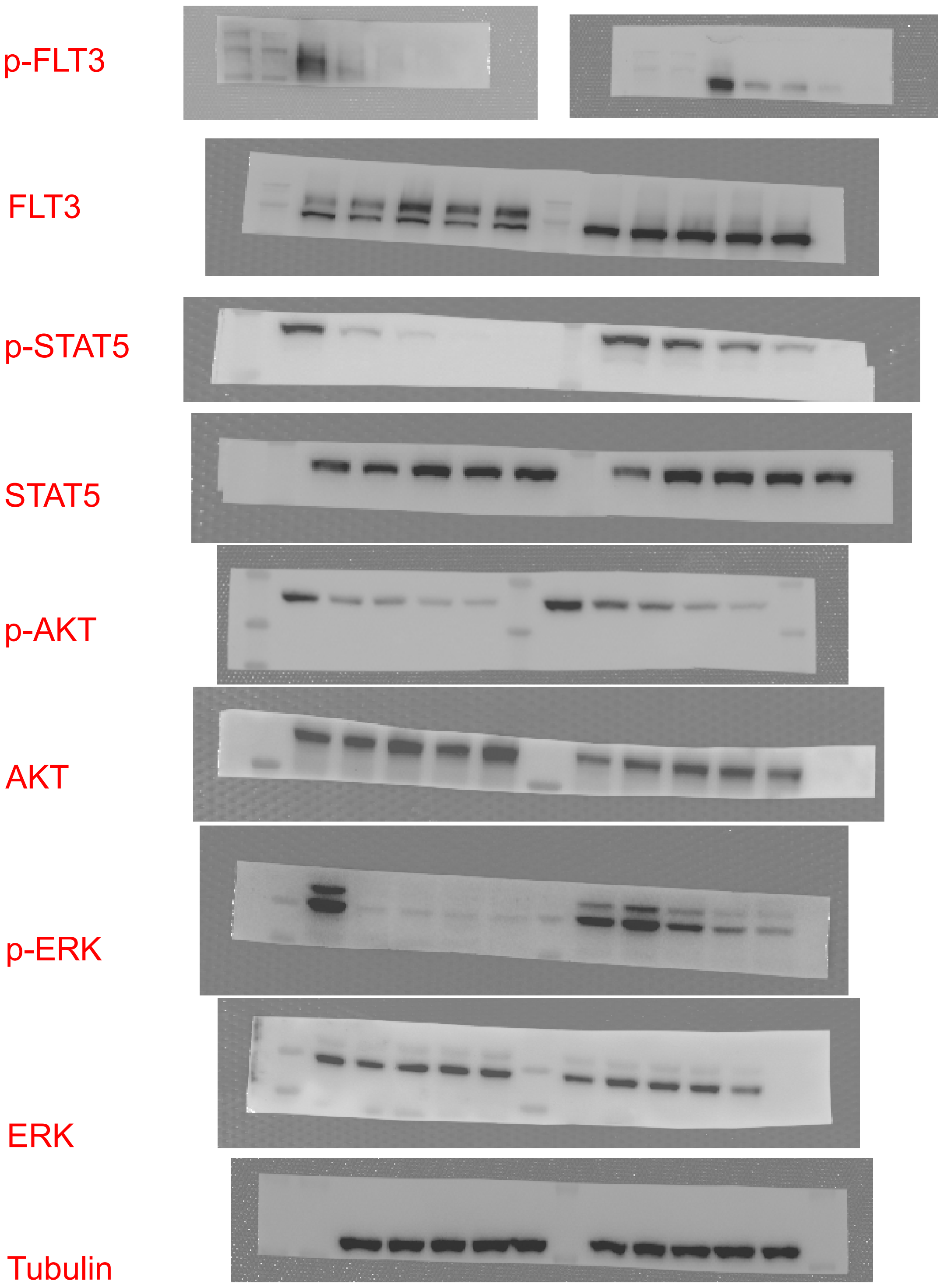

Fig.2 Ba/F3 FLT3-ITD 6h

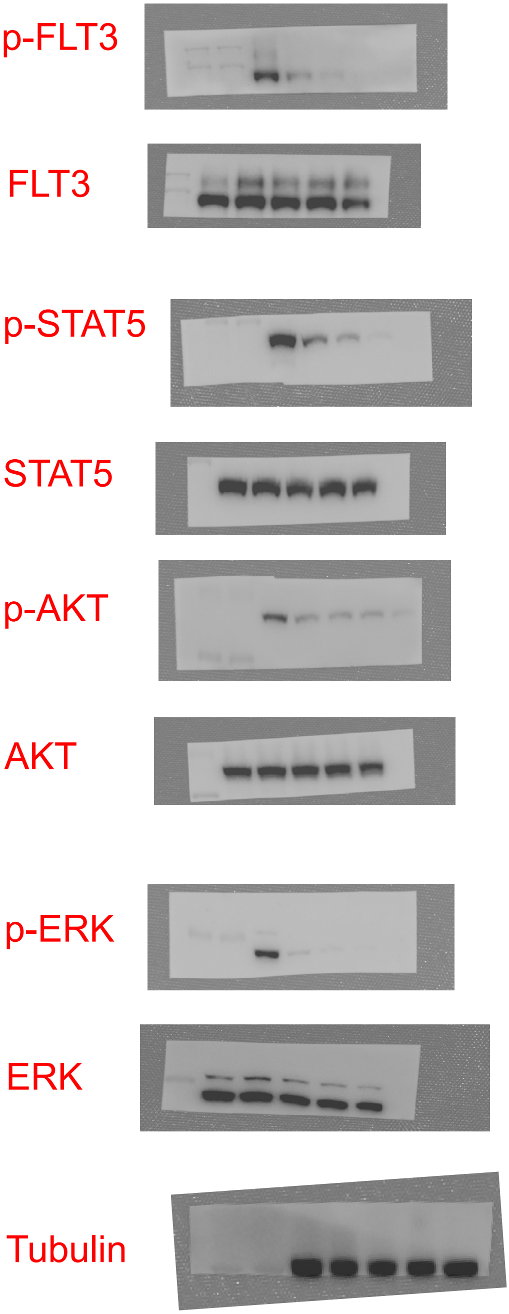

Fig.3C

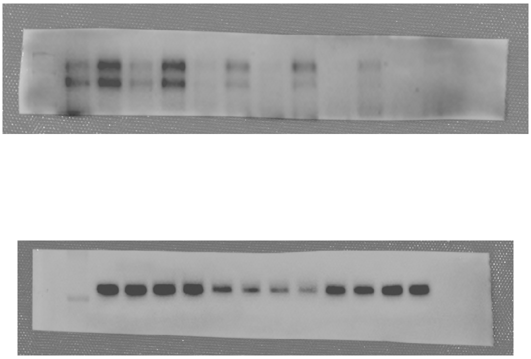

FLT3

Tubulin

Fig.4

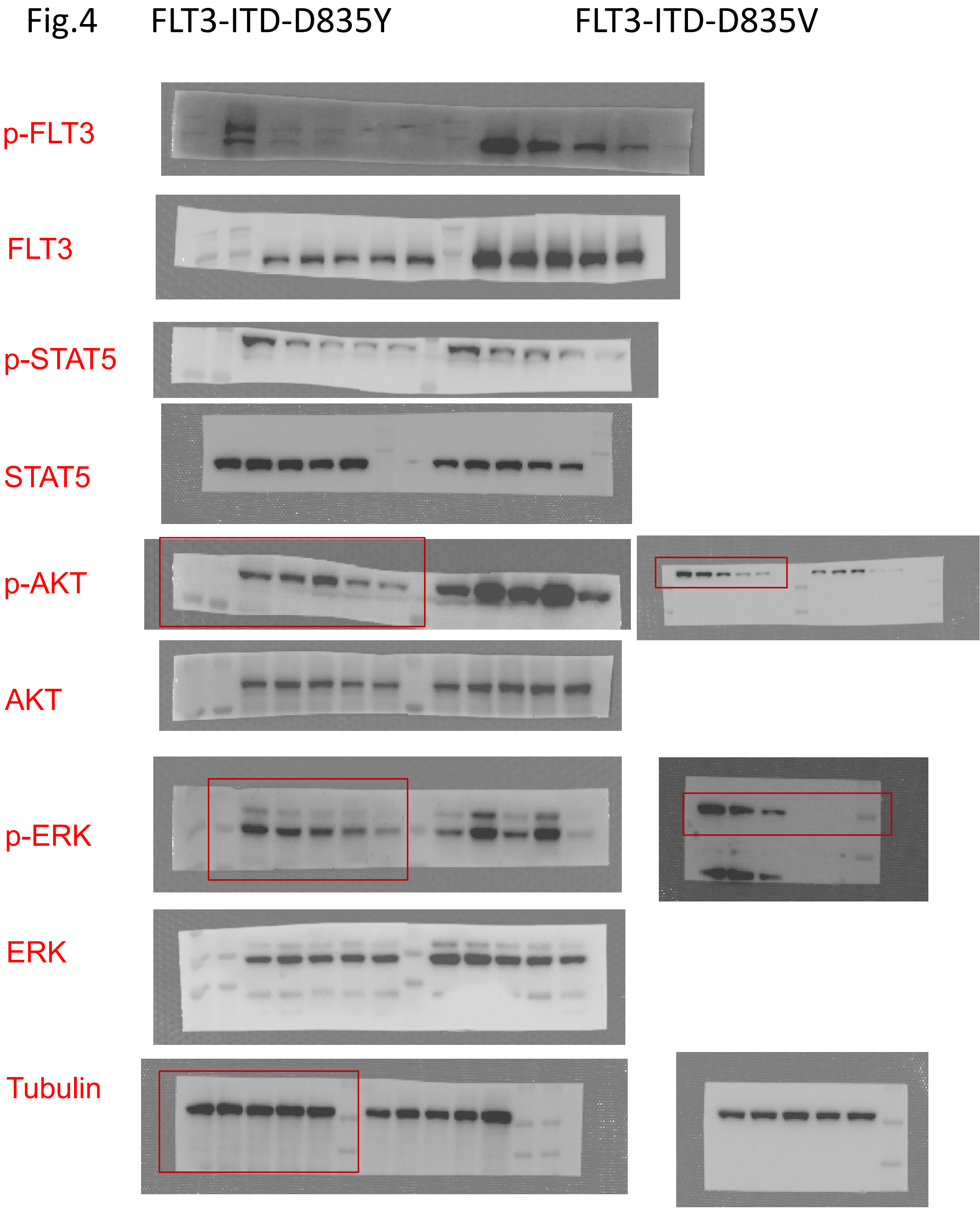
FLT3-ITD-D835Y
FLT3-ITD-D835V
p-FLT3
FLT3
p-STAT5
STAT5
p-AKT
AKT
p-ERK
ERK
Tubulin

Fig.4 FLT3-ITD-Y842C

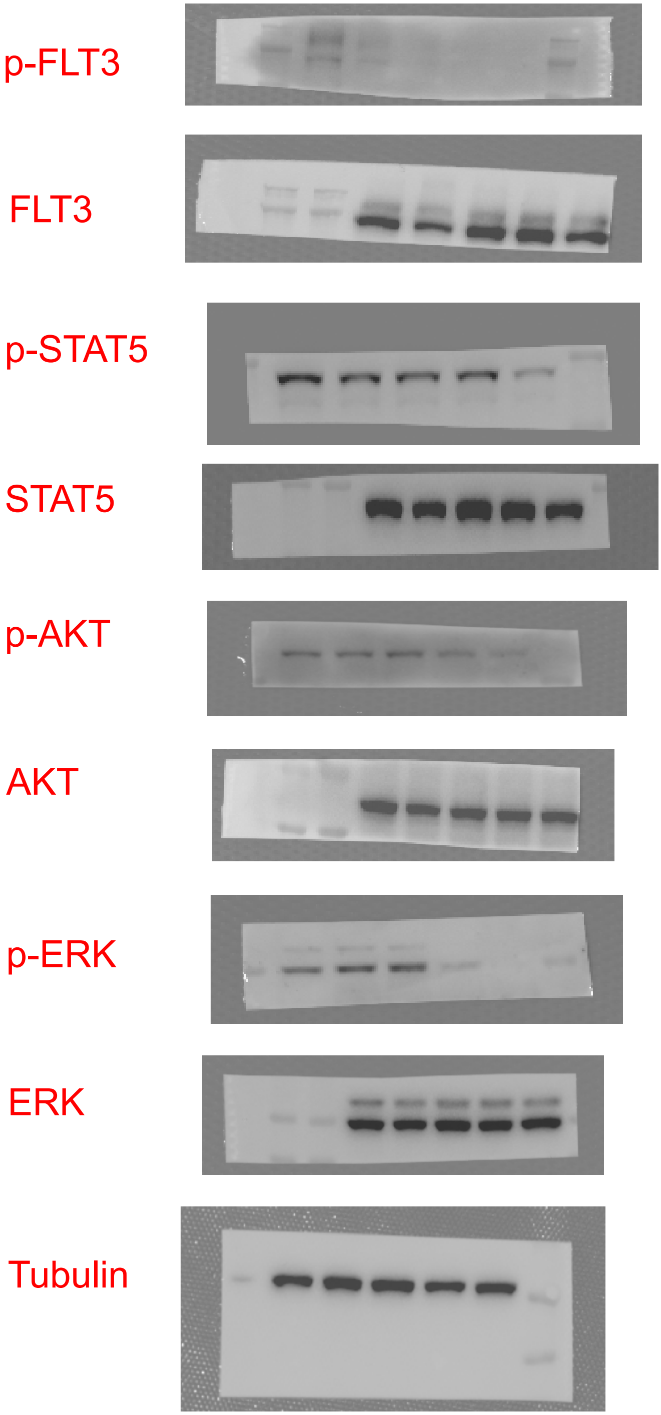

Fig.6D

Fig.6E

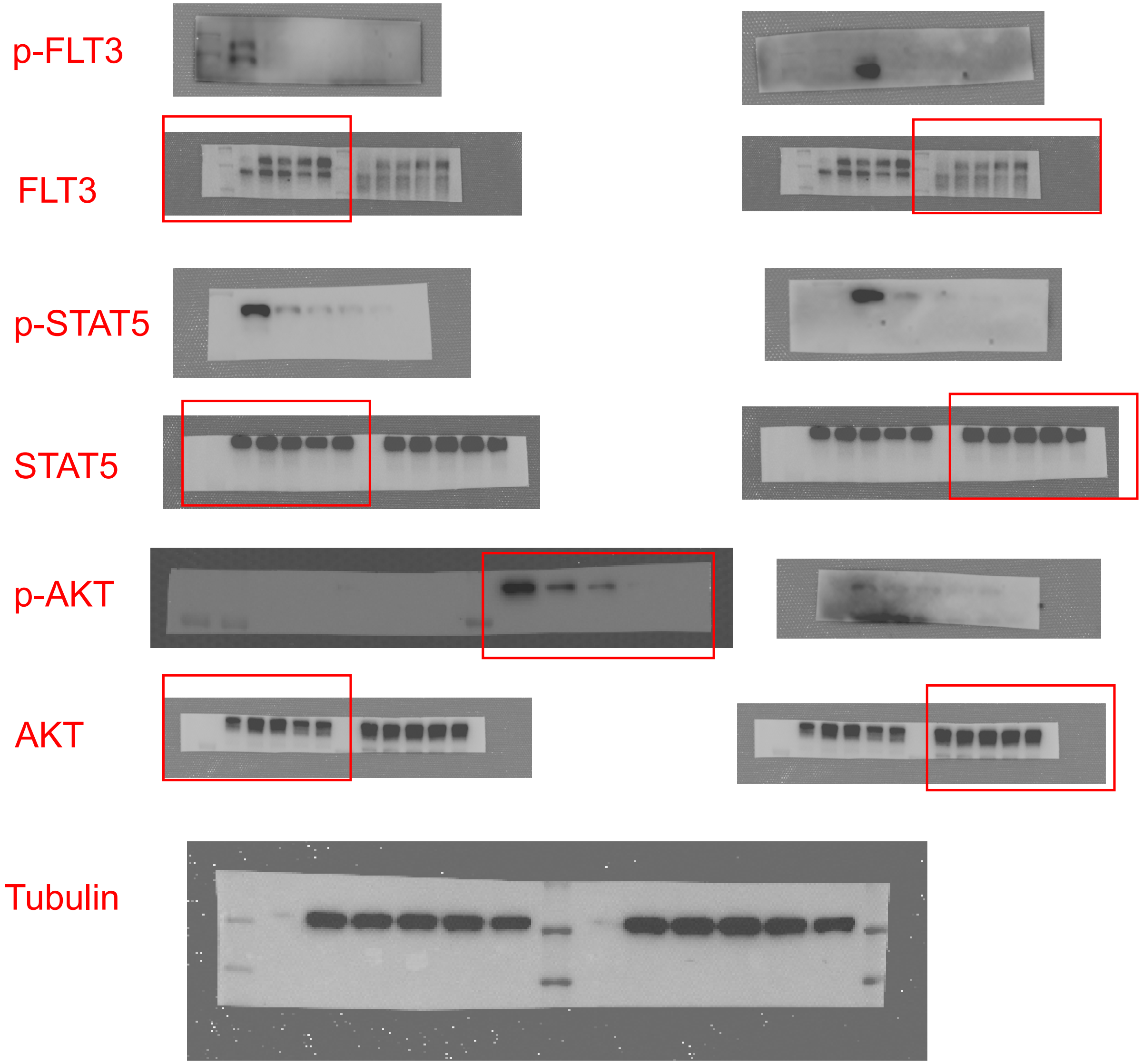

Supplement: Supplementary file 2 — Supplementary Material 2 [file 12964_2024_1729_MOESM2_ESM.pdf]
